# Fronto-Parietal Connectivity Is a Non-Static Phenomenon with Characteristic Changes during Unconsciousness

**DOI:** 10.1371/journal.pone.0087498

**Published:** 2014-01-27

**Authors:** Gisela Untergehrer, Denis Jordan, Eberhard F. Kochs, Rüdiger Ilg, Gerhard Schneider

**Affiliations:** 1 Department of Anesthesiology, Helios Clinic Wuppertal, Witten/Herdecke University, Wuppertal, Germany; 2 Department of Anesthesiology, Klinikum rechts der Isar, Technische Universität München, Munich, Germany; 3 Department of Neurology, Klinikum rechts der Isar, Technische Universität München, Munich, Germany; University of British Columbia, Canada

## Abstract

**Background:**

It has been previously shown that loss of consciousness is associated with a breakdown of dominating fronto-parietal feedback connectivity as assessed by electroencephalogram (EEG) recordings. Structure and strength of network connectivity may change over time. Aim of the current study is to investigate cortico-cortical connectivity at different time intervals during consciousness and unconsciousness. For this purpose, EEG symbolic transfer entropy (STEn) was calculated to indicate cortico-cortical information transfer at different transfer times.

**Methods:**

The study was performed in 15 male volunteers. 29-channel EEG was recorded during consciousness and propofol-induced unconsciousness. EEG data were analyzed by STEn, which quantifies intensity and directionality of the mutual information flow between two EEG channels. STEn was computed over fronto-parietal channel pair combinations (10 s length, 0.5–45 Hz total bandwidth) to analyze changes of intercortical directional connectivity. Feedback (fronto → parietal) and feedforward (parieto → frontal) connectivity was calculated for transfer times from 25 ms to 250 ms in 5 ms steps. Transfer times leading to maximum directed interaction were identified to detect changes of cortical information transfer (directional connectivity) induced by unconsciousness (p<0.05).

**Results:**

The current analyses show that fronto-parietal connectivity is a non-static phenomenon. Maximum detected interaction occurs at decreased transfer times during propofol-induced unconsciousness (feedback interaction: 60 ms to 40 ms, p = 0.002; feedforward interaction: 65 ms to 45 ms, p = 0.001). Strength of maximum feedback interaction decreases during unconsciousness (p = 0.026), while no effect of propofol was observed on feedforward interaction. During both consciousness and unconsciousness, intensity of fronto-parietal interaction fluctuates with increasing transfer times.

**Conclusion:**

Non-stationarity of directional connectivity may play a functional role for cortical network communication as it shows characteristic changes during propofol-induced unconsciousness.

## Introduction

Altered states of consciousness are associated with changes in directional (effective) and functional connectivity within a fronto-parietal network. This is observed as a common phenomenon during anesthesia, vegetative states and sleep [1], [2], [3], [4], [5], [6], [7], [8], [9]. During anesthesia, propofol-induced loss of consciousness has been linked to a preferential inhibition of fronto-parietal feedback connectivity [1], [2], [4], [6], [8], [9], [10]. As feedforward processing is related to primary sensory processing, this indicates that sensory “information is received but not perceived” and identifies disintegration of information processing as the phenomenon behind anesthesia-induced unconsciousness [11]. So far, the neurophysiologic mechanisms behind this disintegration have not been identified.

Recent studies show that functional connectivity of cerebral areas is not a static phenomenon, but shows spontaneous fluctuations over time [12], [13], [14], [15]. Previously, fluctuations of functional connectivity have been thought to reflect changing levels of vigilance, task switching or conscious processing. Today, there is evidence that fluctuating connectivity is an intrinsic phenomenon of brain dynamics that persists even during anesthesia [15]. In macaques, fluctuations of functional connectivity within an attention network have been demonstrated and interpreted as mechanistically important network information [15]. To date, computational approaches design models to explain dynamics of activity, connectivity and their interaction [16], [17]. Condensed, non-static characteristics of network activity and connectivity can be seen as an intrinsic mechanism of the brain to optimize effectiveness of information transfer. Still, the relationship between changes of consciousness and network dynamics is not understood, yet.

However, so far, fluctuations of neuronal network connectivity have been shown for functional connectivity as measured by functional magnetic resonance imaging (fMRI). The current study evaluates whether directional cortical connectivity as measured by electroencephalogram (EEG) may also show fluctuations, but in the range of milliseconds [17]. Based on EEG data, it has been shown that spatiotemporal slow oscillation dynamics could be the reason for inhibited network communication by isolating local cortical networks [18]. Assuming a functional role for network communication, presence and dynamics of directional connectivity are currently analyzed during consciousness and propofol-induced unconsciousness. Thereby it was shown that cortical directional connectivity is a non-static phenomenon with characteristics during consciousness and unconsciousness. This indicates an important relationship between dynamical network connectivity and anesthesia-induced changes of consciousness.

## Materials and Methods

### Clinical Protocol

Fifteen healthy male volunteers (American Society of Anesthesiologists physical status 1 or 2, 21–32 years old) participated after approval by the university's ethics committee (local ethics committee of the medical department of the Technische Universität München, Germany) and informed written consent. The study was conducted in the department of anesthesiology of the Technische Universität München, Germany. Standard monitoring parameters and 29-channel EEG (EASYCAP, Brain Products, Gilching, Germany, international 10-20-system, impedances below 5 kΩ) were continuously recorded with a sampling rate of 5 kHz using BrainAmp (Brain Products GmbH, Gilching, Germany) electroencephalographic amplifier and BrainVision Recorder (Brain Products GmbH, Gilching, Germany) data acquisition software. Propofol was administered by a target controlled infusion pump (target controlled infusion TCI, space infusion pump, BBraun Medical, Melsungen, Germany). Subjects received propofol in stepwise increasing concentrations until loss of consciousness occurred, which was defined clinically when subjects stopped following the investigator's instruction to squeeze his hand. TCI concentration at this point was maintained for 10 minutes to allow equilibration. EEG was recorded without drug application (consciousness, 10 minutes) and during propofol-induced unconsciousness (10 minutes). Basic artifact detection (signal amplitudes exceeding 250 μV) and average reference were performed on EEG recordings.

### EEG Analysis

In existing EEG data of propofol-induced unconsciousness we applied EEG analysis within the framework of an information-theoretic approach allowing a model-free exploration of directed interactions between frontal (process X) and parietal (process Y) electrodes. For this purpose, EEG symbolic transfer entropy (STEn) was calculated [19], [20] to estimate the predictive information transfer between X and Y [21]. Analysis was focussed on the fronto-parietal network to quantify the information exchange between cortical areas with a key role for conscious processing of sensory input and mechanisms linked to unconsciousness [1], [2], [4], [11]. The applicability of STEn for human EEG data has been recently shown [8], [9]. STEn analysis is a nonparametric method based on information theory which allows to detect the preferred direction of cortical interaction between two EEG signals 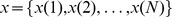 and 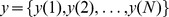 containing N amplitude samples.

**Figure 1:**
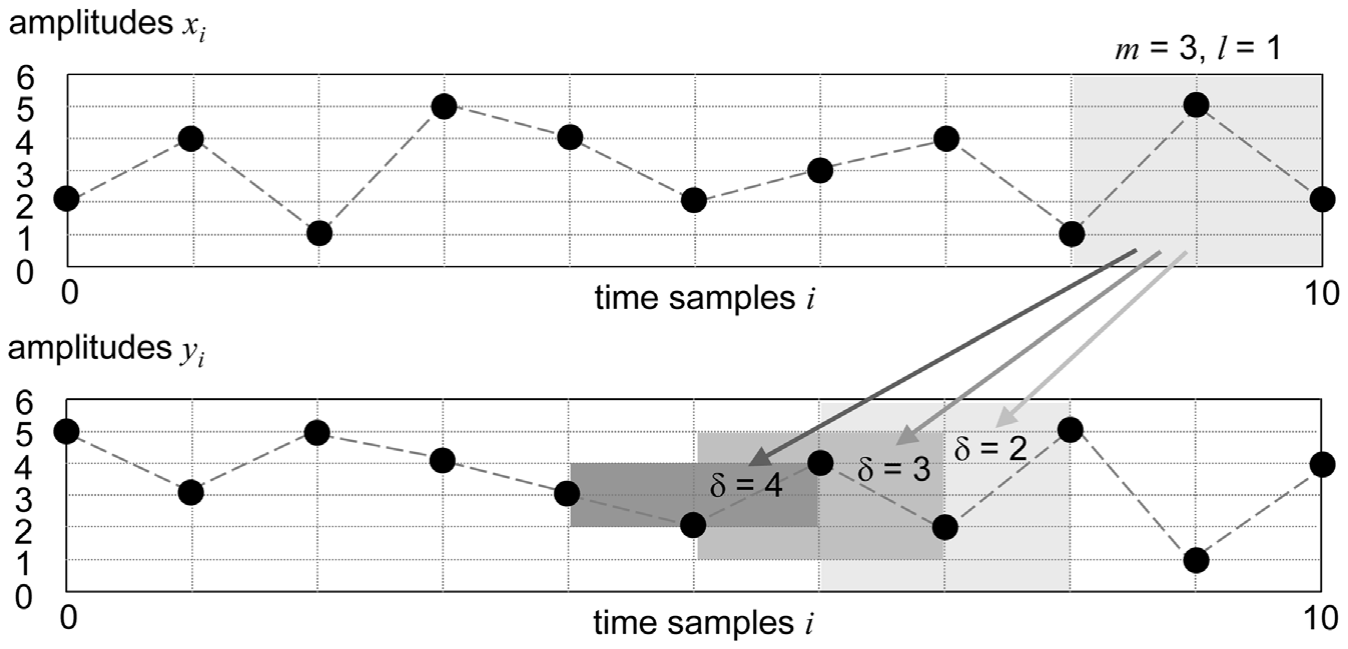
Two exemplary EEG channels x and y showing how 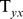 (embedding dimension m = 3, time lag l = 1) indicates the directed interaction from x to y at different transfer times δ = 1, 2, 3.

For calculation, STEn analyzes consecutive sequences 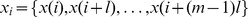 and 
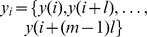
 (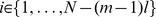) using embedding parameters m (dimension) and l (time lag) in the signals x and y. Then the amplitude order is given by 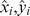. The degree of prediction 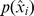 of actual information in x (or y respectively) from past information content in 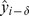 (or 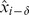 respectively) with respect to a transfer time δ>0 is reflected by a generalized Markov property, where intrinsic prediction within one and the same signal is discarded by “subtracting” the probability 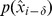 (or 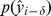 respectively) that 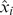 can be explained by 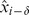 (or 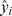 by 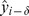 respectively) [21]. Figure 1 shows the meaning of the parameters m, l, δ for the calculation of STEn. In conclusion, calculation of symbolic transfer entropy - which predicts information in signal x from information in signal y - is based on the Shannon entropy, see equation (1).
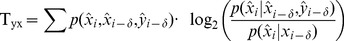
(1)


For frontal (x) and parietal (y) EEG this indicates feedforward parietal → frontal projections between parietal and frontal electrodes (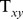 feedback frontal → parietal projections respectively). The sum considers all triplets of order sequences of length m in x, y, where 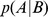 is the conditional probability that A occurs under condition B, and 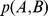 is the joint probability of A and B. The directionality index denoted by STEn quantifies the preferred direction of flow between x and y, see equation (2).
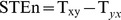
(2)


STEn is expected to attain positive values for unidirectional coupling with x as the driver and negative values for y driving x. Assuming 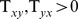, a value STEn  = 0 indicates symmetric bidirectional coupling. STEn was computed using LabVIEW 8.5 (National Instruments, Austin, Texas), where the core algorithms were embedded in C allowing parallel processing on an Intel Xeon based workstation with Windows 7 (Microsoft Corporation, Redmond, Washington).

Frontal and parietal EEG was measured at electrode positions Fp1, Fp2, F3, F4 and P3, P4 (electrode positions according to the international 10-20-system). STEn was computed over fronto-parietal channel pair combinations at consciousness and propofol-induced unconsciousness using one artifact free EEG signal of 10 s length for each subject (total bandwidth of 0.5-45 Hz at 200 Hz sampling frequency). Acording to the signal length criterion *m*! <*N* = 2000 we used *m* = 5 and *l* = 5 in order to provide a suitable deployment of EEG trajectories within the state space during consciousness and propofol-induced unconsciousness [9], [22]. A scanning approach was chosen to detect the transfer time δ with maximum mutual interaction as indicated by 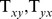. This may reveal effective transmission times for cortico-cortical information transfer between fronto-parietal information processing [21]. Calculations of STEn for different transfer times δ represent the new aspect of the present study (figure 1). Analysis started at 25 ms (δ = 5) [11]. Intervals of 5 ms (δ = 1) were chosen to get close meshed results and 250 ms (δ = 50) were determined as sufficient limit to detect trends of directed interactions. STEn is computed in the time-space which means that a direct comparison to frequency-based signal analysis is not possible. Nevertheless, the applied settings result in a focus on information transfer in time scales within the electroencephalographic β-and lower γ-band band, which is considered to be relevant for long-range intercortical information exchange [23], [24].

### Data Analysis

Values of 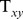 and 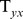 in frontal (4 electrodes) and parietal (2 electrodes) electroencephalogram were averaged. At consciousness and unconsciousness, the transfer time resulting to the first maximum of 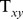 (and 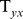 respectively) within 25 ms to 80 ms was identified. Resulting transfer times at consciousness and unconsciousness were compared (Wilcoxon test at threshold p<0.05). Furthermore, 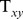 and 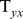 were evaluated during consciousness and unconsciousness (Mann-Whitney-U test at threshold p<0.05).

STEn may indicate spurious interdependencies in the EEG caused by spectral changes. To address this question, surrogates from the electroencephalographic signals were derived by maintaining the spectral amplitudes (i.e. linear properties of mean, variance and auto-covariance structure) and randomising the phase (Fourier based surrogates [2], [25]). In addition to the evaluation on original EEG, 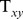, 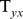 were calculated on 20 surrogates per electroencephalographic signal to test the hypothesis that effects arise from spectral changes as generated by a linear random process (confidence intervals at threshold p<0.05) [2], [9].

## Results

**Figure 2:**
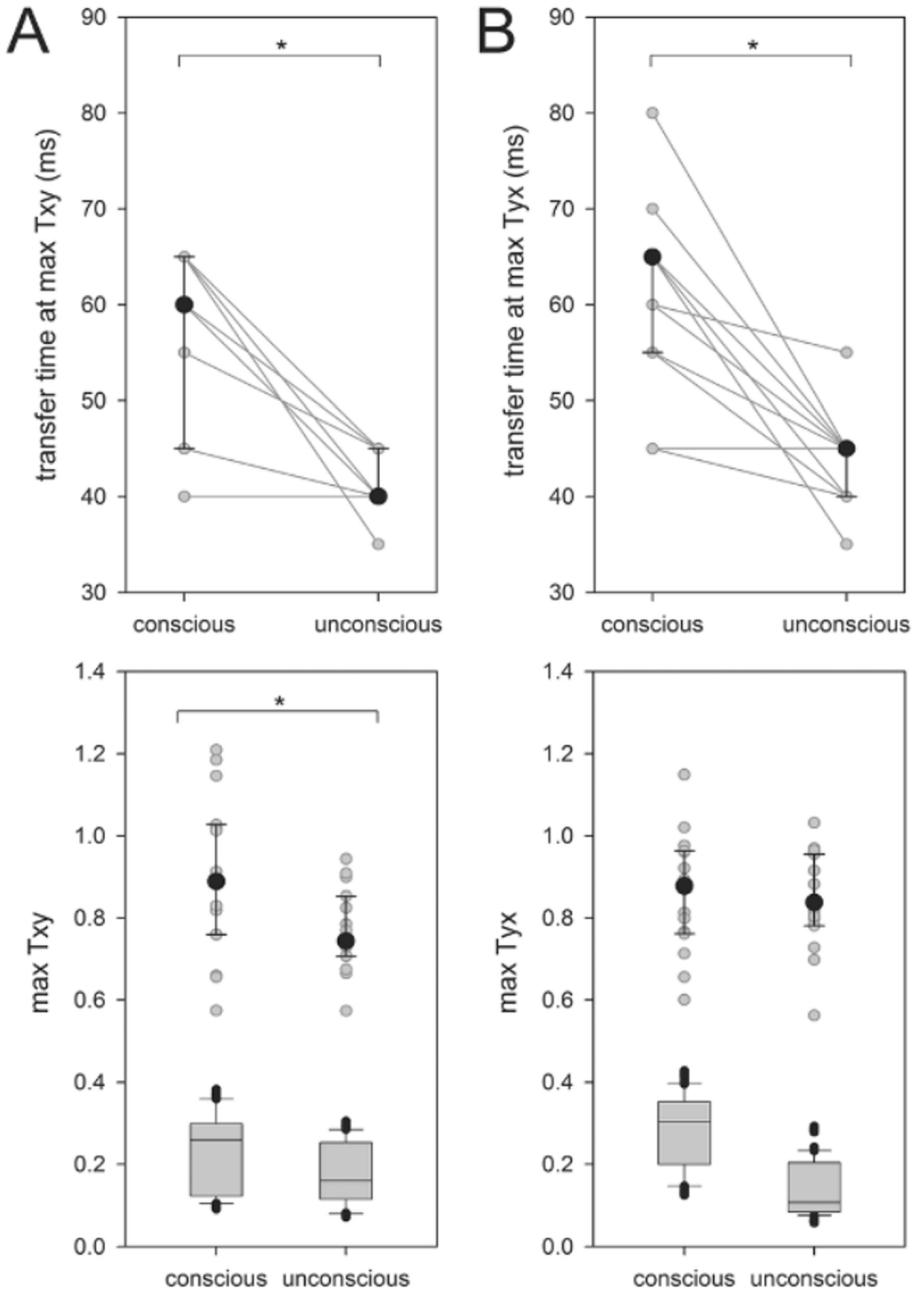
Maximum fronto-parietal EEG connectivity after the first onset (25 ms≤ transfer time ≤80 ms). The upper diagrams (A: feedback 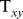; B feedforward 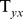) show a significant decrease of transfer time leading to maximum connectivity from consciousness to unconsciousness (grey dots: individual values; black: median with interquartile range; *: p<0.05). The lower diagrams show corresponding values of maximum 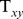 and 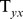
in EEG (grey dots: individual values; black: median with interquartile range; *: p<0.05) and values of the surrogate based 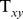 and 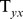 (boxplots).

The entire EEG data set was used for analysis. An onset of transfer entropy beginning at 25 ms transfer time may reflect effective connecting times between brain regions. In particular, the time of the first maximum detected directed interaction decreases from consciousness to unconsciousness (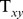: 60 ms to 40 ms, p = 0.002; 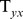: 65 ms to 45 ms, p = 0.001) as shown in figure 2. In contrast, no significant changes of feedback vs. feedforward transfer time are observed at consciousness and at unconsciousness. Furthermore, unconsciousness causes a decrease of maximum feedback interaction 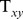 (p = 0.026), while feedforward interaction 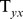 is maintained during unconsciousness (p = 0.417), see figure 2. Surrogate analysis of 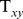, 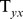 during consciousness and unconsciousness indicates lower interaction than EEG based analysis, both performed at identical transfer time. Values of EEG 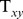, 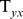 are higher than 95% confidence intervals obtained from surrogate based 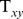, 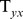 (figure 2).

**Figure 3:**
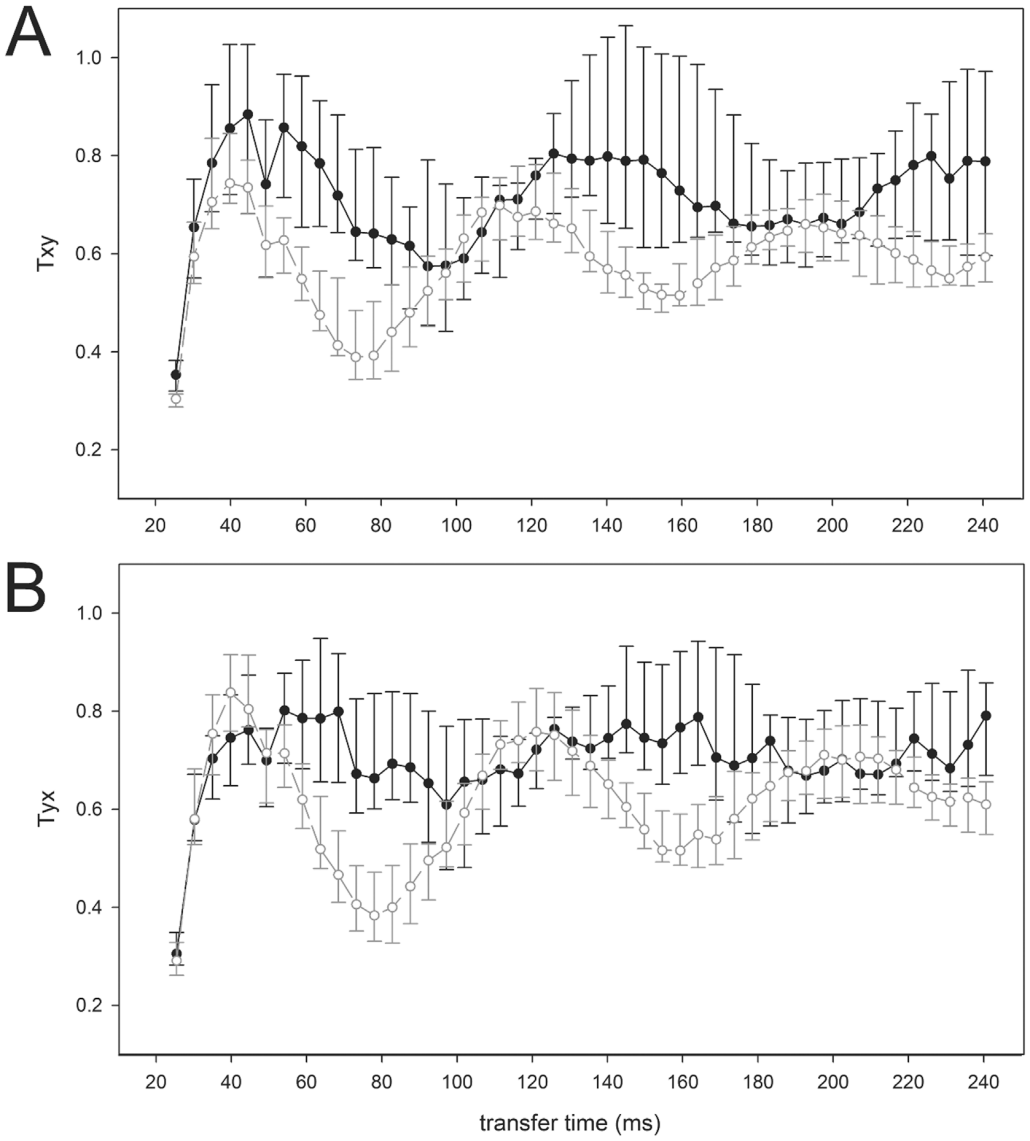
Fluctuating character of directional cortical fronto-parietal connectivity in EEG. Values of directional connectivity (median, 25^th^ and 75^th^ percentile) are illustrated with regard to increasing transfer times from 25 ms to 250 ms (A: feedback 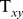; B feedforward 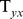; black: conscious; grey: unconscious).

Overall, calculation of STEn for increasing cortico-cortical transfer times shows non-stationarity of directional connectivity: connectivity undulates in the transfer interval from 25 ms to 250 ms (figure 3). Fluctuations of STEn values (i.e. directional connectivity) occur for fronto-parietal feedback and feedforward projections and during both consciousness and unconsciousness. With regard to the present knowledge of feedback breakdown during unconsciousness, figure 3 illustrates that detection of feedback inhibition depends on the transfer time for cortical communication because of the phenomenon of temporary fluctuating fronto-parietal interaction.

## Discussion

The mechanism behind anesthesia-induced loss of consciousness is not a general neuronal inactivation, but a change of dynamic aspects in neuronal network communication [26]. Functionally, consciousness may result from the integration of global neural information, while unconsciousness may be caused by a disruption of this integration capacity of the brain [27]. Directional connectivity within an anterior-posterior network is thought to reflect cortico-cortical causal interactions of information processing. In the last years, intact anterior-posterior feedback connectivity was identified as a key feature of conscious processing: during altered states of consciousness, preferentially feedback projections are inhibited, while feedforward connectivity remains unchanged [1], [2], [4], [6], [7], [8], [9], [26]. The recently presented hub reconfiguration during propofol-induced unconsciousness contains important information to further understand functional mechanisms causing the selective breakdown of feedback connectivity [28].

The current results point out that directional cortical connectivity is a non-static phenomenon not only between but also within certain states of consciousness and unconsciousness. Remarkably, this non-stationarity of directional connectivity differs between consciousness and unconsciousness: during unconsciousness maximum connectivity occurs within significantly shorter time intervals for cortico-cortical information transfer than during consciousness. At last, we show that during unconsciousness, the strength of maximum interaction indicating relevant directional connectivity is maintained for feedforward projections, but decreases for feedback projections, which is in accordance to the present knowledge [1], [2], [4], [6], [7], [8], [9], [26].

One would suggest that during consciousness the content and amount of information is richer and more than during unconsciousness. Then, the detected decrease of transfer time for maximum connectivity during unconsciousness could be due to less information transferred. A reduction of information could be caused by thalamic actions and thalamocortical interactions as propofol also reduces thalamic activity [29], [30]. Although STEn analyses only detect cortico-cortical interactions, the thalamic role for loss of consciousness can not be disregarded. Nevertheless, our results also show that the amount of feedforward connectivity is not reduced during unconsciousness which does not support the theory of thalamic filtering and faster information transfer based on less amount of data.

Faster information transfer during unconsciousness could also be caused by shorter processing time due to fewer activated and thus fewer interconnected cortical substations [26]. Suppressing effects of propofol on neural activity of specific cerebral areas have been shown by positron emission tomography [31]. Among the cortical regions, propofol particularly seems to affect associative areas responsible for the integration of sensory information. If activity is reduced in these regions, their contribution to network connectivity may also be reduced which may result in faster information transfer between parietal and frontal areas, reflected by shorter transfer times for maximum directional connectivity. This theory is consistent with different delays for cortico-cortical communication, which change dependent on the involved cortical areas [11].

Finally, faster information transfer during unconsciousness could also be linked to the currently presented hub reconfiguration during propol-induced loss of consciousness [28]. Lee and colleagues showed an adaptive reconfiguration of functional networks during anesthesia which leads to reduced capacity of information transfer. So, the disrupted parietal hub structure seems to cause the inhibition of feedback-dominant information flow. With a simplified picture, the theory of network hub structure can be described as a road and path network which is given but variably passed, because traffic light circuit changes during different states of consciousness. Concerning this simplified image our result of undulating connectivity and faster information transfer during unconsciousness could reflect faster changes in traffic light circuit and the resulting changes of traffic volume. Thus, changes of parietal and frontal network hub structure during unconsciousness may be associated with the presented changes of non-static connectivity.

Dynamics in brain activity respectively connectivity are thought to play an important role for the functional capacity of the neural network and network communication [16]. In this context undulations of directional connectivity may also reflect a fundamental cerebral mechanism to optimize cortical communication. During consciousness, changes of connectivity have been shown to be linked to attention tasks [32]. So, it seems to be unlikely that during unconsciousness the currently detected fluctuations of connectivity are also related to task-switching. Therefore, non-stationarity may indicate either ongoing, albeit modified cortico-cortical communication during unconsciousness or only intrinsic dynamics of the cortex, similar to fluctuating functional connectivity [15]. The presence of undulations with changed onset of maxima suggests that network communication relies on a certain periodicity of information transfer. Thus, network interaction may be changed if the rhythm of information transfer is altered. For example, loss of consciousness during seizure has been related to over-synchronization between the thalamus and parietal cortex [33]. While synchronization between distant brain regions is thought to be essential for conscious processing [34], abnormal over-synchronization seems to overload cerebral structures and impede them from treating external input. The theory of rhythm-based communication could also be in line with the findings of hub reconfiguration [28].

Calculations of STEn for different transfer times represent the new aspect of the present study. In contrast to other methods analyzing mutual independencies (e.g. Granger causality) non-parametric STEn showed consistent results even in different setups, e.g. embedding parameters, electrode montage [8], [9], [35], [36]. Results of surrogates of the EEG indicate that STEn detects signal characteristics beyond linear randomness and shows effects of propofol which are related to cortical interaction mechanisms.

### Limitations

Further studies with different anesthetics and altered states of consciousness are required to clarify some open points. Unconsciousness was induced by propofol. Therefore, it remains an open question, whether the detected results are a propofol-specific phenomenon or reflect characteristics of unconsciousness in general. The small number of subjects and low spatial resolution of the fronto-parietal network analyzed in EEG represent further limitations. Method inherent issues can not be fully excluded. Estimation of transfer times as performed in the present analysis may also include intrinsic prediction within one area [17]. Because this does not represent a dominant issue for the present data we estimated STEn in concordance with previous works [2], [9].

In conclusion, our results indicate that cortical information transfer is not a static process but fluctuates periodically. Fluctuations of directional connectivity seem to reflect an intrinsic phenomenon of the brain to energetically optimize cortical communication. Characteristically, fluctuations are changed during propofol-induced unconsciousness towards faster information transfer. At the same time, the amount of parieto-frontal feedforward projections is not reduced during unconsciousness, while fronto-parietal feedback interactions are reduced. Both the inhibition of feedback connectivity and the altered fluctuation of information transfer during propofol administration are proposed as functional correlates of loss of consciousness.
